# Therapeutic effects of statins against lung adenocarcinoma via p53 mutant-mediated apoptosis

**DOI:** 10.1038/s41598-019-56532-6

**Published:** 2019-12-31

**Authors:** Cheng-Wei Chou, Ching-Heng Lin, Tzu-Hung Hsiao, Chia-Chien Lo, Chih-Ying Hsieh, Cheng-Chung Huang, Yuh-Pyng Sher

**Affiliations:** 1 Graduate Institute of Biomedical Sciences, China Medical University, Taichung, 404 Taiwan; 2 Chinese Medicine Research Center, China Medical University, Taichung, 404 Taiwan; 3 Research Center for Chinese Herbal Medicine, China Medical University, Taichung, 404 Taiwan; 4 Center for Molecular Medicine, China Medical University Hospital, Taichung, 404 Taiwan; 5 Division of Hematology/Medical Oncology, Department of Medicine, Taichung Veterans General Hospital, Taichung, 407 Taiwan; 6 Department of Medical Research, Taichung Veterans General Hospital, Taichung, 407 Taiwan; 7 Department of Radiation Therapy and Oncology, Shin Kong Wu Ho-Su Memorial Hospital, Taipei, 111 Taiwan

**Keywords:** Targeted therapies, Non-small-cell lung cancer

## Abstract

The *p53* gene is an important tumour suppressor gene. Mutant *p53* genes account for about half of all lung cancer cases. There is increasing evidence for the anti-tumour effects of statins via inhibition of the mevalonate pathway. We retrospectively investigated the correlation between statin use and lung cancer prognosis using the Taiwanese National Health Insurance Research Database, mainly focusing on early-stage lung cancer. This study reports the protective effects of statin use in early-stage lung cancer patients regardless of chemotherapy. Statin treatments reduced the 5-year mortality (odds ratio, 0.43; *P* < 0.001) in this population-based study. Significantly higher levels of cellular apoptosis, inhibited cell growth, and regulated lipid raft content were observed in mutant *p53* lung cancer cells treated with simvastatin. Further, simvastatin increased the caspase-dependent apoptotic pathway, promotes mutant p53 protein degradation, and decreased motile activity in lung cancer cells with *p53* missense mutations. These data suggest that statin use in selected lung cancer patients may have clinical benefits.

## Introduction

Statins, which target the rate-limiting enzyme in cholesterol biosynthesis (3-hydroxy-3-methylglutaryl coenzyme A [HMG-CoA] reductase), are used as lipid-lowering agents to reduce cardiovascular events in patients at risk for atherosclerotic vascular disease^1^. They also reduce long-term coronary heart disease events and related mortalities in patients without risk of cardiovascular disease^2^. Previous experimental results demonstrated the HMG-CoA-dependent anti-tumour effects of statins *in vitro* and *in vivo*, and HMG-CoA-independent effects have been reported when statins are used as broad-spectrum agents in disease pathways, including inflammation, immunomodulation, and angiogenesis^3^. However, the efficacy of statins in cancer prevention and protection in population-based studies has been controversial^4^. These inconsistent findings are likely related to cancer type, sample size, follow-up periods, or genetic background.

Lung cancer is a leading cause of cancer mortality worldwide^5,6^, with disease stage being the major factor influencing mortality. Preventing metastasis and enhancing treatment response are the key factors for prolonging patient survival. In recent population-based studies, long-term statin use reduced overall lung cancer mortality^7,8^.

Statins can be divided into two categories, including lipophilic and hydrophilic statins. A population-based cohort study revealed that lipophilic simvastatin is more efficacious at reducing rates of cancer-specific mortality than other hydrophilic statins^9^. Statins might also enhance the therapeutic effects and overall survival in lung cancer patients receiving epidermal growth factor receptor-tyrosine kinase inhibitor (EGFR-TKI) therapy^10^. However, conflicting results from randomized trials showed no superiority in the statin-combined treatment group^11–13^.

Mechanisms underlying the reduction in cancer mortality after statin treatment are not clearly understood. However, in a previous breast cancer study, sterol biosynthesis genes were highly expressed in patients with a *TP53* (p53) mutation, implicating the mevalonate pathway as a possible therapeutic target^14^. There is a high percentage of lung cancer patients with p53 mutations; approximately 46–62% of patients with lung adenocarcinoma have p53 mutations^15^. Most (61%) p53 mutations are missense mutations within the DNA binding domain and correlate with poor outcomes^16^. In addition, a different spectrum of mutations is observed in patients who smoke than in patients who have never smoked. HMG-CoA reductase inhibitors modify the mevalonate pathway and attenuate the smoking-induced carcinogenesis pathway^17^. The p53 gene plays a critical role in tumour suppression and modulates several key cellular functions, including senescence, apoptosis, autophagy, and metabolic reprogramming processes^18^. Here, we investigated the impact of p53 mutations on the treatment effects of simvastatin in lung adenocarcinoma. We found that simvastatin is more toxic in lung adenocarcinomas that harbour *TP53* mutations, providing a possible therapeutic strategy in lung cancer treatment.

## Results

### Statin treatment decreases early lung cancer mortality

**Figure 1:**
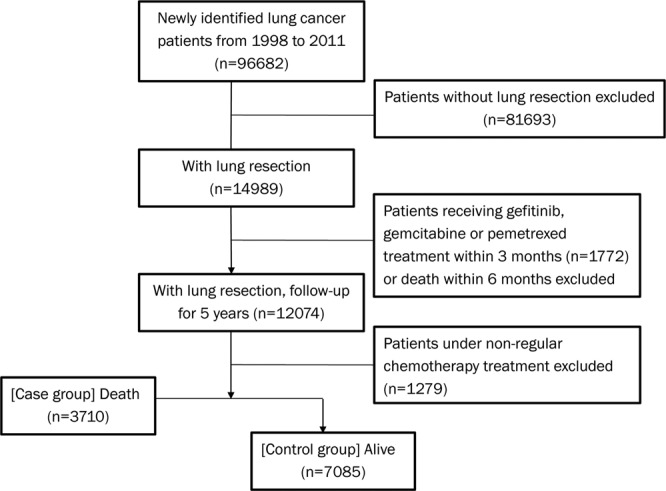
Flowchart describing lung cancer patient cohort and patient selection.

To examine whether statin use confers a protective effect that reduces mortality in lung cancer patients, we included patients from the National Health Insurance Research Database (NHIRD) between 1998 and 2011. The median follow-up time was 5 years. A total of 96682 patients with newly diagnosed lung cancer were enrolled in this study. We focused on the early-disease stage, attributable to the heterogeneity of the patient population. To identify patients at the early stage, we established several criteria to exclude late-stage patients as described in the methods sections. A total of 10795 early lung cancer patients were included for further analysis (Fig. 1). The exposure period for statin use was defined as more than 4 weeks of statin treatment after lung cancer diagnosis to death or end of follow-up. The chemotherapy cohort and non-chemotherapy cohort are listed in Table 1. The comparison of incidence density of 5-year mortality between patients with statin use or not is reported in Table 2. Among all patients analysed, statin use conferred protective effects in lung cancer patients, with a reduced 5-year mortality (odds ratio, 0.43; 95% confidence interval [CI], 0.37–0.49; *P* < 0.001). These patients were mainly diagnosed during the early stage of cancer rather than locally advanced or metastatic disease, as we only enrolled patients with lung resection without further treatment for metastatic disease according to the above criteria.

**Table 1:** Comparison of baseline characteristics between patients with regular C/T and without C/T among lung cancer patients with resection.

Variables	Regular C/T (n = 1356)		Non-CT (n = 9439)		p-value
	n	(%)	n	(%)	
Age					<0.001
<50	315	(23.2)	1118	(11.8)	
50–64	648	(47.8)	3332	(35.3)	
≥65	393	(29.0)	4989	(52.9)	
Sex					0.702
Women	589	(43.4)	4048	(42.9)	
Men	767	(56.6)	5391	(57.1)	
CCI score					<0.001
≤3	398	(29.4)	4320	(45.8)	
4–6	154	(11.4)	2533	(26.8)	
≥7	804	(59.3)	2586	(27.4)	

C/T, chemotherapy; CCI, Charlson comorbidity index.

**Table 2:** Comparison of incidence densities of 5-year mortality between patients with drugs and without drugs among lung cancer patients with resection (all patients).

Variables	Number (Y/N)	Univariate model			Multivariate model		
		OR	(95% CI)	*P*	OR^‡^	(95% CI)	*P*
Statin	1391/9404	0.43	(0.37–0.49)	<0.001	0.47	(0.40–0.55)	<0.001
Atorvastatin	596/10199	0.33	(0.26–0.42)	<0.001	0.34	(0.27–0.44)	<0.001
Fluvastatin	165/10630	0.32	(0.21–0.50)	<0.001	0.41	(0.25–0.66)	<0.001
Lovastatin	97/10698	0.40	(0.24–0.68)	<0.001	0.73	(0.41–1.30)	0.285
Pravastatin	113/10682	0.43	(0.27–0.70)	<0.001	0.75	(0.44–1.27)	0.283
Rosuvastatin	341/10454	0.25	(0.18–0.34)	<0.001	0.23	(0.16–0.33)	<0.001
Simvastatin	229/10566	0.45	(0.32–0.62)	<0.001	0.65	(0.45–0.94)	0.024

^‡^Adjusted for observation time, sex, age, and Charlson comorbidity index (CCI) score; Y, yes; N, No.

The Charlson comorbidity index (CCI) is a method of predicting outcomes and risk of death from many comorbid diseases according to their potential influence on mortality and is a valid prognostic indicator for mortality^19^. We further analysed these data using a multivariate model by adjusting for observation time, sex, age, and CCI score; protective effects for reducing the 5-year mortality were observed for most statins in the multivariate model, except lovastatin and pravastatin, probably attributable to fewer cases (Table 2). To investigate whether the protective effects of statins remain in patients receiving chemotherapy, these patients were separated into two groups based on the chemotherapy schedule, including a regular chemotherapy (C/T) and non-C/T group. Table 3 demonstrates that the protective effects of statins were still observed in non-C/T patients. Taken together, statin use had a protective effect in early-stage lung cancer patients regardless of chemotherapy.

**Table 3:** Comparison of incidence densities of 5-year mortality between patients with drugs and without drugs among lung cancer patients with resection by C/T status.

Variables	Regular C/T (n = 1356)				Non-C/T (n = 9439)			
	Number (Y/N)	OR	(95% CI)	*P*	Number (Y/N)	OR	(95% CI)	*P*
Statin	141/1215	0.49	(0.31–0.76)	0.002	1250/8189	0.47	(0.40–0.56)	<0.001
Atorvastatin	55/1301	0.27	(0.12–0.64)	0.003	541/8898	0.35	(0.27–0.45)	<0.001
Fluvastatin	16/1340	NA	NA	NA	149/9290	0.47	(0.29–0.77)	0.003
Lovastatin	5/1351	0.64	(0.06–6.33)	0.703	92/9347	0.73	(0.40–1.33)	0.298
Pravastatin	9/1347	0.23	(0.03–1.98)	0.181	104/9335	0.85	(0.49–1.47)	0.571
Rosuvastatin	43/1313	0.44	(0.20–1.00)	0.049	298/9141	0.21	(0.14–0.32)	<0.001
Simvastatin	15/1341	0.69	(0.19–2.48)	0.568	214/9225	0.64	(0.44–0.95)	0.025

Adjusted for observation time, sex, age, and Charlson comorbidity index (CCI) score. NA, not available; C/T, chemotherapy; Y, yes; N, no.

Another population-based study showed that lipophilic statins are associated with a more significant reduction in cancer-specific mortality and all-cause mortality, particularly when treated with simvastatin^9^. Further, simvastatin induced anti-proliferative and anti-migration effects in lung cancer cell lines as well as in a mouse model^20–22^. Therefore, we further investigated the effects of simvastatin in lung cancer cell lines.

### Simvastatin inhibits lung cancer cell growth

**Figure 2:**
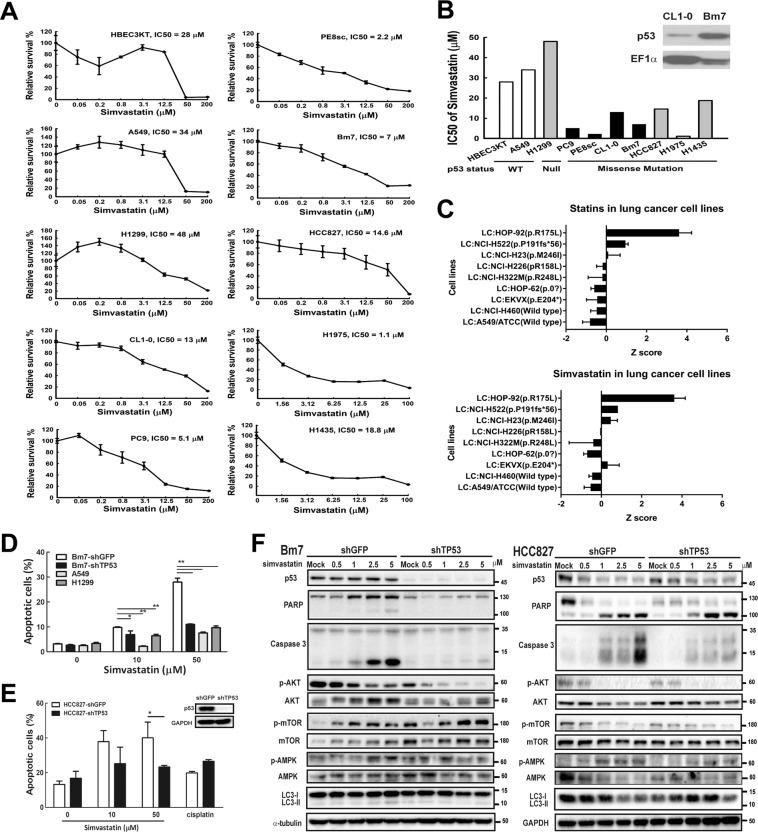
Simvastatin increases cytotoxicity in lung cancer cells. (**A**) Relative survival (%) in lung cancer cells treated with simvastatin for 48 h using 3-[4,5-dimethylthiazol-2-yl]-2,5 diphenyl tetrazolium bromide (MTT) assays is shown. (**B**) The half-maximal inhibitory concentration (IC_50_) of simvastatin is summarized, and western blots of p53 in low-invasive CL1-0 and high-invasive Bm7 cells are shown with elongation factor 1 alpha (EF1α) used as a loading control. (**C**) Z score of statins as well as simvastatin in lung cancer cell lines from NCI-DTP database, z score > 0 for sensitive and <0 resistant. (**D**) Apoptotic H1299 (null p53), A549 (wild type p53), Bm7-shGFP (mutant p53), and Bm7-shTP53 (knock-down p53) cells treated with simvastatin were detected using flow cytometry, *P < 0.05 and **P < 0.01. (**E**) Apoptotic HCC827-shGF*P* (mutant p53) and HCC827-shTP53 (knock-down p53) cells treated with simvastatin and cisplatin were detected using flow cytometry, *P < 0.05 and **P < 0.01. (**F**) Western blots of indicated proteins involved in apoptosis and autophagy in both Bm7 and HCC827 cells with control (shGFP) and p53 knockdown (shTP53) treated with simvastatin is shown. MDM2, murine double minute 2; AKT, serine–threonine kinase; PARP, poly (ADP-ribose) polymerase; mTOR, mammalian target of rapamycin; WT, wild type. Full-length blots/gels are presented in Supplementary Fig. 1.

From the above population-based case-control study, we show that statins provide benefits to dyslipidaemia patients with lung cancer. To investigate the therapeutic effects of statins in lung cancer cells, we first determined the cytotoxicity of simvastatin in several lung cancer cell lines, including immortalized normal lung epithelial cells (HBEC3KT); lung cancer A549, H1299, PC9, HCC827, H1975 and H1435 cells; and an established lung cancer cell line (PE8sc) obtained from the pleural effusion of a lung cancer patient. Relative cell viability was measured by setting untreated cells in each cell line to 100% and calculating the half maximal inhibitory concentration (IC_50_) dosage in cells treated with simvastatin for 2 days. We discovered a wide range of IC_50_ values among these lung cancer cells when treated with simvastatin (Fig. 2A). Notably, normal bronchial epithelial cells (HBEC3KT), lung cancer cells with wild type (WT) p53 (A549), or null p53 (H1299) required higher doses of simvastatin to reach a 50% cytotoxic effect (IC_50_ near/over 30 μM). In contrast, lung cancer cells with a p53 missense mutation exhibited an significantly lower IC_50_ compared to wild type or null. Moreover, we performed a cytotoxicity assay in CL1-0 (low invasive) parental cells and the Bm7 (highly invasive) sub-line; these two cell lines contain a p53 mutation (R248W) resulting in higher levels of p53 in Bm7 cells than in CL1-0 cells (Fig. 2B) despite being derived from the same patient. In addition, Bm7 cells had a nearly 2-fold lower IC_50_ (7 μM) than the parental CL1-0 cells (13 μM). These data suggest that simvastatin has higher therapeutic effects in lung cancer cells containing p53 mutations. We also extracted data from web-based tool of the Developmental Therapeutics Program (DTP) of the National Cancer Institutes (NCI) (https://discover.nci.nih.gov/cellminer/). In the NCI-60 panel, z score was determined through the lung cancer cells with sensitive (z score > 0) and resistant (z score < 0) results^23^. Several lung cancer cell lines with mutant p53 also response to statins or simvastatin but not in wild type cells (Fig. 2C)^24^. Therefore, statins seem to provide a therapeutic choice for lung cancer with mutant p53.

### Simvastatin induces apoptosis and has little effect on autophagy in lung cancer cells

To investigate whether decreased cell survival associated with simvastatin treatment is accompanied by apoptotic features, we examined the distribution of apoptotic cells using annexin-V/propidium iodide (PI) staining in cell lines with a different p53 status, including null (H1299), wild type (A549), mutant p53 (Bm7-shGFP, HCC827-shGFP), and knock-down (Bm7-shTP53, HCC827-shTP53) cells. After treatment with simvastatin (0, 10, or 50 μM) for 48 h, a significant increase in apoptotic cells was observed in the mutant p53 (Bm7-shGFP) cell line in a dose-dependent manner (Fig. 2D). However, only a slight increase in apoptotic cells in the p53 wild type and null cells was detected. Compared with mutant p53 cell lines, the effect of simvastatin-induced apoptosis was abrogated by knocking down mutant p53 (Bm7-shTP53) to a comparable level in p53 wild type or null cancer cells (Fig. 2D). In another mutant p53 lung cancer cell line, HCC827 (p53 V218del), simvastatin induced cell apoptosis and its apoptotic effect were reduced after knocking down mutant p53 as well. Compared to conventional chemotherapy, cisplatin induced similar cell apoptosis rate regardless of p53 status (Fig. 2E). This demonstrates that simvastatin-induced cell apoptosis was increased in lung cancer cells with mutant p53. We then determined whether simvastatin induces the caspase-dependent apoptotic pathway in lung cancer cells containing a p53 mutation. In Bm7 cells with a p53 mutation (shGFP) treated with different concentrations of simvastatin for 48 h, the levels of cleaved poly (ADP-ribose) polymerase (PARP), a key protein involved in apoptosis, and cleaved caspase-3 were dose-dependently higher than those in the mock controls (Fig. 2F). In contrast, this phenomenon was not observed in p53-knockdown cells (shTP53), suggesting p53 mutations increase cell apoptosis in lung cancer cells treated with simvastatin (Fig. 2F). Similar results of the higher cleaved caspase-3 levels were also shown in control cells treated with simvastatin than p53 knockdown HCC827 cells (Fig. 2F).

The Akt signalling pathway has an important role in modulating cell growth and survival^25^. However, we found that simvastatin moderately reduced the level of phospho-Akt in p53 mutant control cells; the decreasing trend of phospho-Akt was also found in p53 knockdown cells. Therefore, Akt signalling is not likely related to rescue apoptosis in p53 knockdown cells treated with simvastatin (Fig. 2F).

Since fluvastatin, a type of statin drug, has been reported to induce autophagy in lung cancer cells through the p53-AMPK-mTOR pathway via fluvastatin-induced AMPK phosphorylation and mTOR (Ser-2448) dephosphorylation^26^, we next examined the signalling pathway involved in autophagy in cancer cells treated with simvastatin. AMPK phosphorylation only slightly increased after treatment with simvastatin but there was no significant difference between mutant p53 and knockdown cells. In addition, no obvious difference of microtubule-associated protein 1 A/1B-light chain 3 (LC3-II), a marker of autophagy, was observed after treatment with high doses of simvastatin in Bm7 or HCC827 cells. Phospho-mTOR wasn’t affected by the mutant status of p53, suggesting that mTOR signalling was not significantly regulated by the mutant 53 status. Therefore, simvastatin-mediated cytotoxicity of lung cancer cells with mutant p53 may be dependent on cell apoptotic pathways, with little effect on autophagy.

### Simvastatin decreased lipid rafts in lung cancer cells with p53 mutations

**Figure 3:**
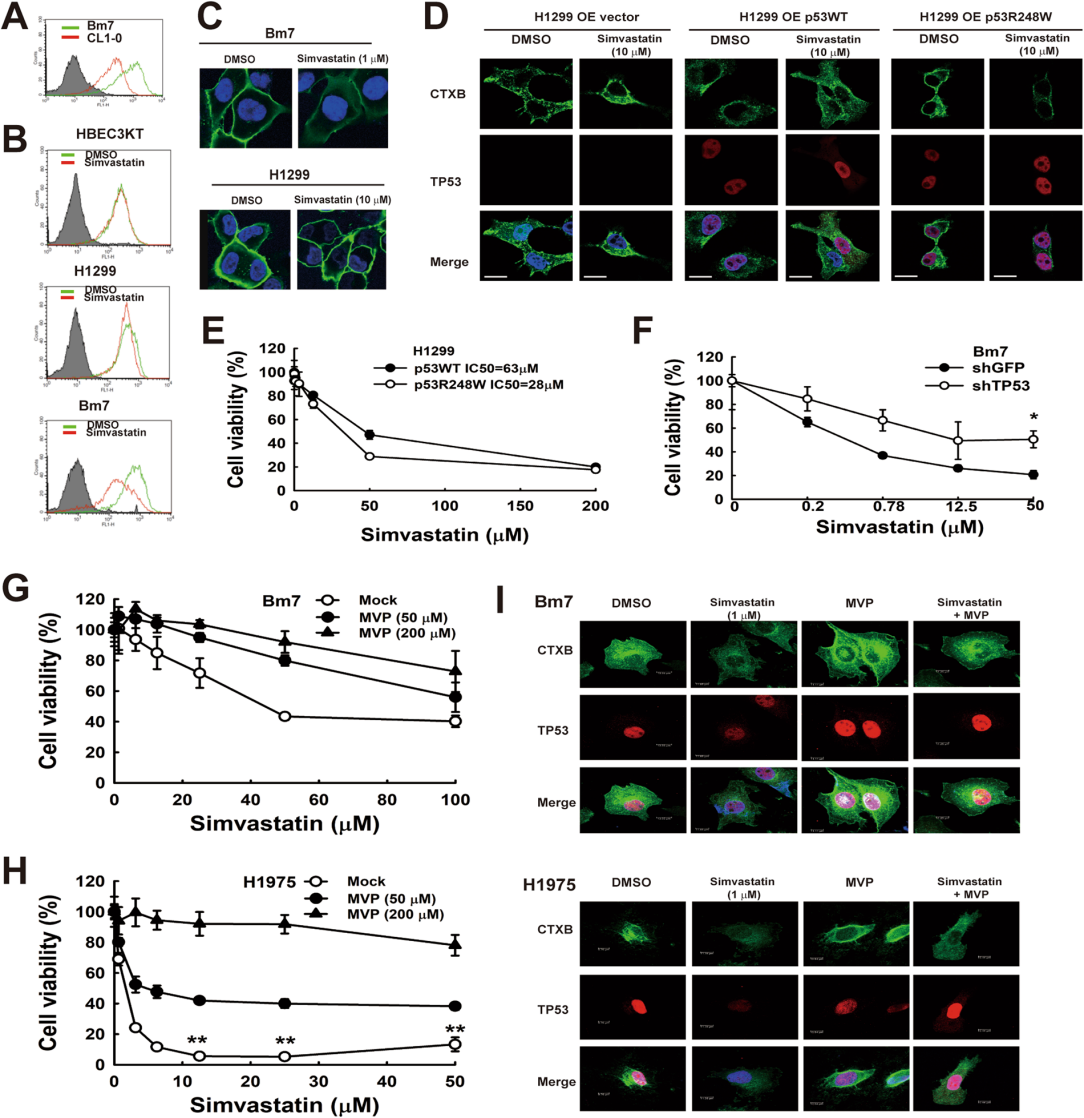
Simvastatin reduces lipid raft content and cell viability in lung cancer cells containing p53 mutations. (**A**) Lipid rafts were fluorescence-labelled with CTXB and detected using flow cytometry in CL1-0 and Bm7 cells. (**B**) Lipid raft levels in lung cancer cells were measured after simvastatin treatment. (**C**) Lipid rafts in Bm7 and H1299 lung cancer cells treated with simvastatin were stained with fluorescence-labelled CTXB and imaged using confocal microscopy. (**D**) Lipid rafts were measured in H1299 cells transiently transfected with plasmids of vector, p53WT, and p53R248W under simvastatin treatment. (**E**) Cell viability was determined in H1299 cells transiently transfected with p53WT or p53R248W plasmids under simvastatin treatment. The half maximal inhibitory concentration (IC_50_) is shown. (**F**) *TP53* knockdown increased cell viability in Bm7 cells treated with simvastatin. (**G**,**H**) Simvastatin induced cytotoxic effects in Bm7 and H1975 cells could be rescued by MVP supplementation. (**I**) Decrease of lipid raft by simvastatin could be reversed by MVP supplementation. DMSO, dimethyl sulfoxide; MVP, mevalonate-5-phosphate.

Cholesterol plays an essential role in maintaining membrane integrity and is critical for lipid raft formation in cell membranes. High levels of lipid rafts can serve as a platform for enhancing receptor mediated-cell transformation and metastases^27^. Thus, we tested whether simvastatin influences the lipid raft content of lung cancer cells, which in turn modulates cell viability and metastasis. First, we measured lipid rafts in cells with cholera toxin B (CTXB) using a flow cytometry assay^28^. Signals representing lipid raft staining were stronger in Bm7 cells than parental CL1-0 cells (Fig. 3A). After simvastatin treatment, the fluorescent signal quickly declined in Bm7 lung cancer cells. In contrast, signals in simvastatin-insensitive cells, including HBEC3KT and H1299 cells, were slightly decreased during simvastatin treatment (Fig. 3B). A decrease in lipid rafts on the membrane of Bm7 cells treated with 1 μM simvastatin was detected using immunofluorescence staining; however, no differences were observed in H1299 cells treated with 10 μM simvastatin (Fig. 3C). We also transiently transfected vector, wild type p53, and mutant p53 (R248W) into the p53 null H1299 cells and determined the effect of p53 on lipid rafts. After simvastatin treatment, a significant decrease in immunofluorescence staining of lipid rafts (CTXB) was detected in the mutant p53 plasmid transfected cells, and CTXB staining remained at high levels in vector or wild type p53-overexpressed cancer cells (Fig. 3D). These results indicate that simvastatin reduces the presence of lipid rafts on p53 mutant lung cancer cell membranes.

### Simvastatin has greater cytotoxic effects in lung cancer cells with p53 mutations

To further investigate whether overexpression of *TP53* mutations increases cytotoxicity in lung cancer cells treated with simvastatin, we transiently transfected WT *TP53* and R248W mutants into p53 null H1299 cells and then measured cell viability under simvastatin treatment (Fig. 3E). H1299 cells overexpressing p53 mutations were more sensitive to simvastatin treatment than the WT p53 cells, with IC_50_ values of 28 μM and 63 μM, respectively. In addition, cell viability under simvastatin treatment was higher after knocking down endogenous mutant p53 in Bm7 cells than in control Bm7 cells (Fig. 3F). Therefore, the p53 mutant status of lung adenocarcinoma cells affects the cytotoxic potency of simvastatin. Further, we found significantly higher simvastatin-induced inhibition in lung cancer cells with p53 mutations.

To validate the effects of statin through mevalonate pathway in mutant p53 cells, we treated Bm7 (p53 R248W) and H1975 (p53 R273H) cells with simvastatin and the supplement of mevalonate-5-phosphate (MVP). With MVP supplement, the cytotoxic effects of simvastatin in mutant p53 lung cancer cells can be rescued (Fig. 3G,H). This has demonstrated that simvastatin’s cytotoxic effect in mutant p53 lung cancer cells was through the inhibition of mevalonate pathway downstream signalling pathway. In CTXB staining of both Bm7 and H1975 cells, simvastatin reduced lipid raft, and MVP enhanced the presence of lipid raft staining (Fig. 3I). Also, with the supplement of MVP, simvastatin induced reduction of lipid raft was at least partially recovered in these mutant p53 cell lines (Fig. 3I). These results reveal that the change of lipid raft by simvastatin is mainly through mevalonate pathway of mutant p53 lung cancer cells.

### Simvastatin suppresses the motile activity of lung cancer cells

**Figure 4:**
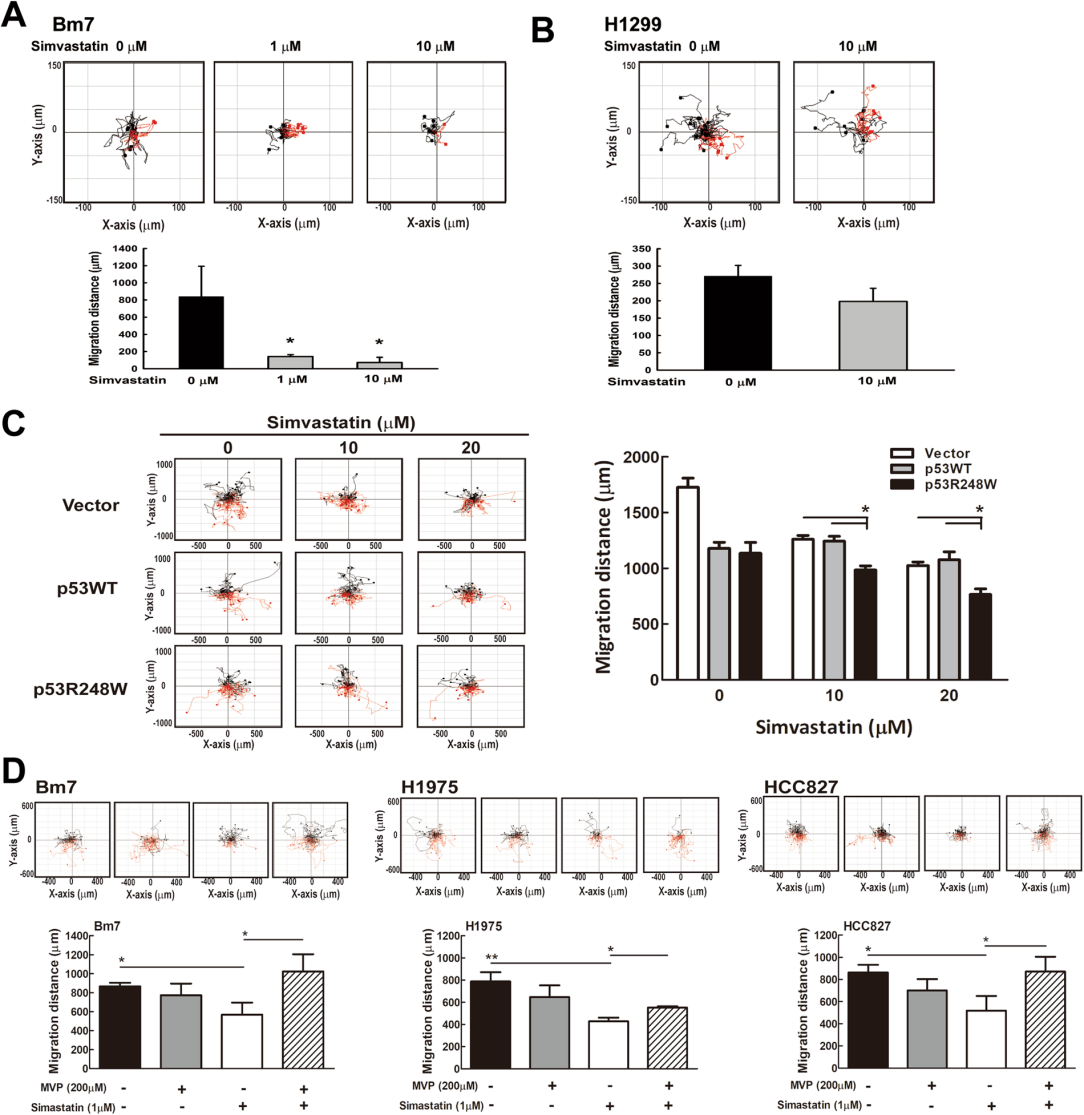
Simvastatin reduces cell migration in lung cancer cells with p53 mutations. (**A**) The migration distance for Bm7 lung cancer cells treated with simvastatin was measured using time-lapse video microscopy (top) and then quantified (bottom). Error bars: SD from three independent experiments; *P < 0.05. (**B**) The migration distance for H1299 lung cancer cells treated with simvastatin using time-lapse video microscopy as in (**A**) is shown. (**C**) The migration distance for H1299 cells transiently transfected with plasmids of vector, p53WT, and p53R248W under simvastatin treatment was measured via time-lapse video microscopy (left). The quantified migration distance is shown (right), *P < 0.05. (**D**) The migration distances for Bm7 (R248W), H1975 (R273H), and HCC827 (V218del) cell lines under simvastatin treatment with or without MVP supplement were measured, *P < 0.05. Cell migration to the right is shown in red, and to the left is shown in black.

To assess whether simvastatin treatment affects the motile activity of lung cancer cells, we measured the migration distance (motile activity) for lung cancer cells treated with simvastatin using time-lapse video microscopy. The accumulative migrated distance substantially decreased in Bm7 cells treated with a low concentration (1 μM) of simvastatin, whereas it was slightly decreased in H1299 cells treated with 10 μM simvastatin (Fig. 4A,B). To validate this result, we transfected the H1299 cell line with wild type and mutant p53 (R248W) plasmids and then treated with simvastatin. We also observed a significant and dose-dependent decrease in migration after simvastatin treatment in mutant p53 cells (Fig. 4C). Furthermore, we compared the motile activities in Bm7 (R248W), H1975 (R273H), and HCC827 (V218del). In these cell lines with mutant p53, we all observed significantly reduction of migration distance after treated with simvastatin as well as at least partially rescued by MVP (Fig. 4D). These results demonstrate that simvastatin influences migration in lung cancer cells, particularly in those cells with p53 missense mutations and could be reversed by mevalonate pathway rescue.

### Simvastatin increases mutant p53 protein degradation

**Figure 5:**
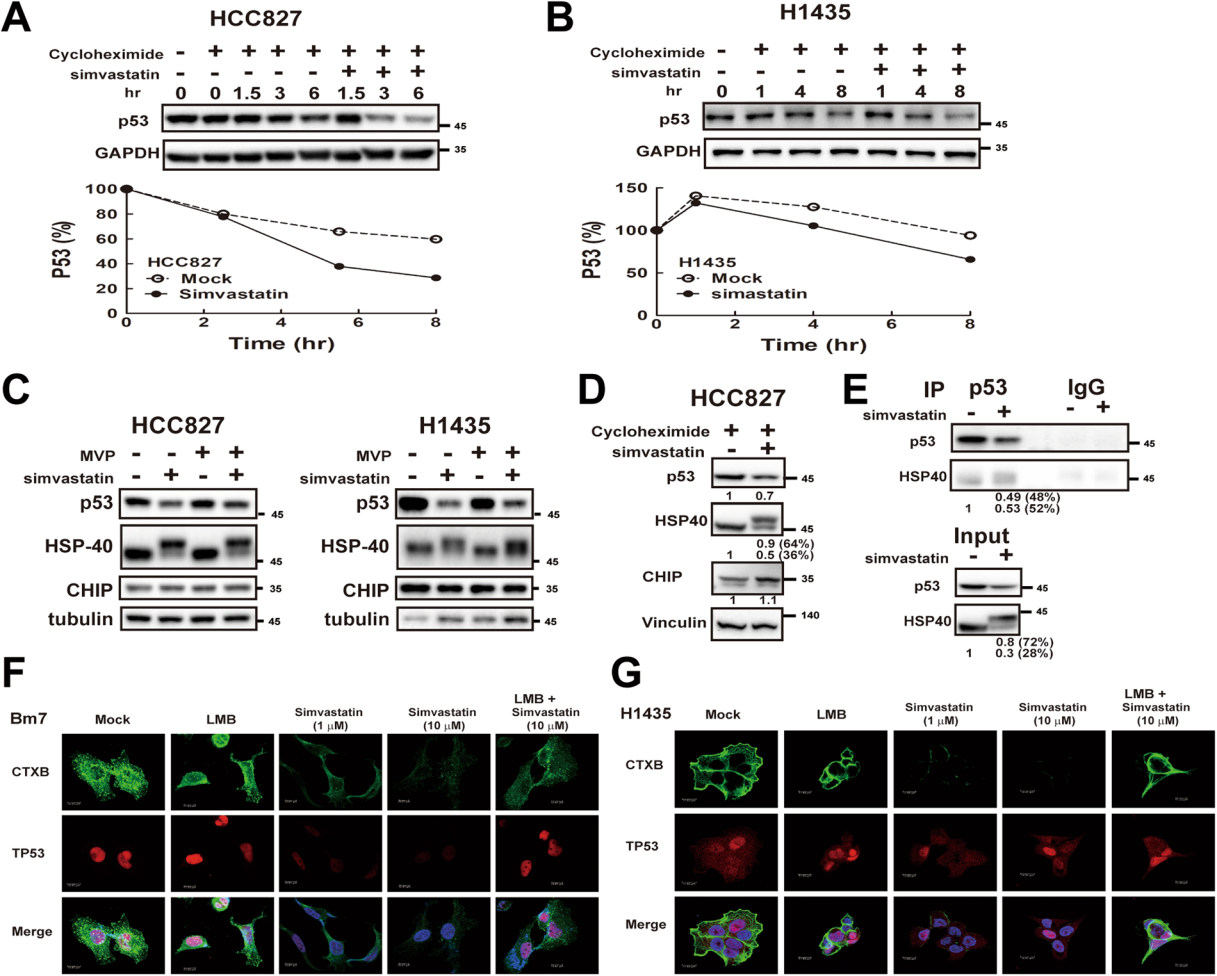
Simvastatin induces mutant p53 degradation and nuclear export. (**A**,**B**) Western blots of indicated proteins and quantification in the presence of cycloheximide (50 nM) and simvastatin treatment are shown for HCC827 and H1435 cell lines. (**C**) Western blots of indicated proteins treated with simvastatin and with or without the supplementation of MVP are shown. Full-length blots/gels are presented in Supplementary Fig. 1. (**D**) A western blot of indicated proteins under the treatment of simvastatin is shown. Numbers beneath the panel indicate the amount of protein relative to the control. Full-length blots/gels are presented in Supplementary Fig. 1. (**E**) Co-immunoprecipitation of western blot is shown to determine the interaction of HSP-40 and p53. Full-length blots/gels are presented in Supplementary Fig. 1. (**F**,**G**) Mutant p53 cell line Bm7 (**F**) and H1435 (**G**) treated with simvastatin and LMB (50 nM) under the image of confocal microscopy.

Since mutant p53 stabilization is crucial for the gain-of-function activity, lung cancer cell lines (HCC827 and H1435) with different mutant p53 status (V218del and C141W, respectively) were treated with simvastatin to determine whether simvastatin enhances the mutant p53 degradation. By treating with cycloheximide to reduce the protein synthesis, simvastatin indeed increases the protein degradation rate of mutant p53 proteins in HCC827 and H1435 lung cancer cell lines (Fig. 5A,B). To investigate whether statins are attributable to decreased binding of mutant p53 to Hsp40/DNAJA1, further increasing the interaction between mutant p53 and the E3 ligase, C terminus of Hsc70-interacting protein (CHIP), for promoting p53 degradation^29^, first, we investigate the levels of mutant p53, HSP-40, and CHIP proteins in lung cancer cells treated with simvastatin. We found that p53 proteins were decreased, and accompanied with increased level of higher molecular weight HSP-40 in simvastatin treatment, whereas the CHIP proteins remained constant (Fig. 5C). MVP supplement in simvastatin treatment partially restored the levels of mutant p53 proteins and the regular molecular weight HSP-40 (Fig. 5C), suggesting that mutant p53 protein expression is associated with the regular molecular weight HSP-40. With cycloheximide treatment, it showed a similar phenomenon that simvastatin induced the high molecular weight HSP-40 expression and reduced the p53 expression (Fig. 5D). It suggests that the high molecular weight HSP-40 is probably modified from the regular molecular weight HSP-40 instead of isoform expression. Moreover, despite the higher level of higher molecular weight HSP-40 in the simvastatin treatment group, mutant p53 proteins interacted mainly with the regular molecular weight HSP-40 in simvastatin treatment as the detection in no simvastatin group (Fig. 5E). It suggests that regular molecular weight HSP-40 proteins are likely to maintain the stability of mutant p53. Next, we investigated whether simvastatin increased nuclear export of mutant p53 for facilitating the mutant p53 degradation. Indeed, simvastatin reduces the levels of nuclear p53 and the lipid raft (Fig. 5F,G). However, leptomycin B (LMB), a nuclear export inhibitor, rescued the simvastatin-mediated p53 nuclear export and also restored the lipid rafts in Bm7 and H1435 lung cancer cells (Fig. 5F,G), suggesting nuclear p53 contributes on the maintenance of lipid rafts. These findings demonstrate that simvastatin enhances the mutant p53 protein degradation and induces nuclear export of mutant p53 for further degradation.

## Discussion

In this study, we demonstrate that statins exert anti-tumour effects during tumour progression using clinical and cell-based evidence. We aimed to determine the long-term effects of statin usage in early lung cancer patients using the Taiwan NHIRD as a source. The NHIRD provides limited information on tumour stage or the EGFR status of patients. Also, there is no p53 status of tumour provided in this database; however, we identified early-stage lung cancer patients by applying exclusion criteria. Among this population, statins conferred a protective effect by reducing the 5-year mortality in those who received statin treatment. Moreover, similar clinical benefits were observed in early resection patients and those receiving adjuvant chemotherapy. Regardless of the p53 mutant status of the population, we demonstrate the protective effects of using statins in lung cancer patients.

The p53 tumour-suppressor gene is the most common mutated gene in most cancer types^30^. Mutant p53 proteins become stable and frequently accumulate in tumour cells^31^. Diverse p53 mutations result in different functional consequences; however, p53 remains an attractive target gene, and several drugs have shown a certain degree of therapeutic efficacy including restoring WT p53 function, disrupting the gain-of-function response partners, degrading mutant p53, and inhibiting p53-mediated survival pathways^32^. However, promising therapeutic agents for most p53-mutant cancers remain elusive or are still under study.

In lung cancer cells, the mevalonate pathway has different transcriptional alterations between different p53 mutations, leading to potential therapeutic targets for specific p53 mutations^33^. Moreover, the anti-tumour effects of statins are reportedly attributable to modulation of pro-inflammatory and oxidative stress-related tumourigenic events^34^. Statins also inhibit tumour metastasis by triggering WT p53-dependent autophagy^26^. Further, statins cause apoptosis in cancer cells via Akt signalling-dependent down-regulation of survivin and the Ras/Raf/mitogen-activated protein kinase kinase (MEK)/extracellular-signal regulated kinase (ERK) signalling cascade^25,35^. These results suggest that statins have tumour suppressive effects in both normal and mutant p53 cells by regulating different signalling pathways.

In our study, simvastatin was less cytotoxic toward non-transformed or benign lung tumour cells. *In vitro* studies have shown that lipid rafts influence receptor-mediated transformation and metastatic abilities of cells, and cholesterol is a major component of lipid rafts. Herein, a significant decline in lipid rafts is observed in p53 mutant cell lines treated with simvastatin. Further, treatment with simvastatin also decreased the migration distance, especially in p53 mutant lung cancer cell lines, which might influence micro-metastases of lung cancer cells *in vivo*. The effects of simvastatin in mutant p53 lung cancer cells mainly act through regulating the mevalonate pathway.

In our study, we used R248W mutated cells previously describing as having a gain-of-function for tumour progression and mutation hotspot^36^. Indeed, p53 mutations are present in about 50% of non-small cell lung carcinomas (NSCLC), with almost 80% of them being missense mutations^37^. Thus, statins are potentially protective in nearly half of NSCLC cases.

Statins exert a weak effect on WT or null p53 lung cancer cells, such as H1299; however, cell survival and cell migration are likely to be moderately decreased at the high dose level used in our study. These results indicate that simvastatin, a cholesterol-synthesis inhibitor, has greater inhibitory effects in p53 mutant cells than in WT p53 and null cells by means of its gain-of-function properties, which upregulate the mevalonate/sterol biosynthesis pathway and act as a therapeutic target^38^.

Without simvastatin treatment, we found ectopic expression of either p53 WT or R248W mutant in p53 null H1299 cells can significantly reduce the migration distance compared with the vector group (Fig. 4C). The finding is similar to previous report that deletion of p53 gene resulted in significant increase of migration rate which might be related to Rho signalling pathway^39^. It is likely due to gain of function in p53 WT or mutant in reducing migration without the simvastatin treatment. Moreover, in vector group, simvastatin seems to significantly influence the cell migration and that may be due to critical roles of lipid raft in cancer cell migration^27^.

Our results have demonstrated that statin treatment results in degradation and nuclear export of mutant p53 in different mutant p53 cell lines. These effects can be rescued by supplementation of MVP. In previous study, the protective effects of statins are likely attributed to decreased binding of mutant p53 to Hsp40/DNAJA1, and further increasing the interaction between mutant p53 and the C terminus of Hsc70-interacting protein (CHIP). This phenomenon activates a ubiquitin ligase-mediated degradation process, which only occurs in p53 conformational mutants^29^.

Using a population-based study, we found that the protective effects of statins associated with advanced-stage lung cancer patients are also observed in early-stage disease patients. Further, the p53-mutant status of lung cancer patients might be a potential predictor for the therapeutic effects of statins in clinical treatment.

## Materials and Methods

### Study population

This study used medical records from the Taiwanese National Health Insurance Research Database (NHIRD), covering 23 million patients between 1998 and 2011. This database includes more than 99% of the entire population of Taiwan, with comprehensive clinical visit records for each insured patient. Further, the database includes inpatient and outpatient dates, diagnostic codes (International Classification of Disease, Revision 9, Clinical Modification [ICD-9-CM]), and prescription records. This study was approved by the institutional review board of Taichung Veterans General Hospital and all research was performed in accordance with relevant guidelines/regulations.

### Study subjects

Study subjects were selected from patients aged ≥20 years who were diagnosed with lung cancer (ICD-9-CM code 162) between 1998 and 2011. Patients who received lung resection were enrolled in a follow-up period up to 5 years (14,989 patients). Patients that received gefitinib, gemcitabine, or pemetrexed within 3 months (1,772 patients) were excluded because these agents were not covered by national insurance at that time. Patients who died within 6 months (1,143 patients) were also excluded. To further identify patients who received complete adjuvant chemotherapy, we excluded patients without regular chemotherapy treatment (1,279 patients, defined as less than 4 instances of chemotherapy within 6 months). Patients were then divided into two groups. One group received adjuvant chemotherapy (regular chemotherapy group), and the other group of patients did not (i.e. they were only followed up and are designated the no chemotherapy group).

### Cell lines

Human HBEC-3KT (p53 wild type), A549 (p53 wild type), H1299 (p53 null), PC9 (p53 R248Q), PE8sc (p53 Q97L), CL1-0 (p53 R248W), Bm7 (p53 R248W), HCC827 (p53 V218del), H1975 (p53 R273H) and H1435 (p53 C141W) lung adenocarcinoma cell lines were used in this study. Immortalized normal lung epithelial cells (HBEC-3KT) were kindly provided by Dr. John D. Minna^40^. The Bm7 cell line is a brain-metastatic clone derived from a highly metastatic sub-line, F4, which has a higher invasion capability than its parental cell line, CL1-0^41^. Primary cultured PE8 cells were established from the pleural effusion of a lung cancer patient under approval from the institutional review board of China Medical University Hospital (CMUH) with written informed consent. CMUH committee has approved the experiments, including any relevant details. All experiments were performed in accordance with relevant guidelines and regulations. Lung cancer cells were confirmed to be free of mycoplasma and were authenticated via DNA typing (Genelabs Life Science, Taipei, Taiwan).

### Cell viability assay

3-[4,5-Dimethylthiazol-2-yl]-2,5 diphenyl tetrazolium bromide (MTT) assays were conducted to determine cytotoxicity after treatment with simvastatin. The IC_50_ was calculated using the CalcuSyn program (Biosoft, Cambridge, United Kingdom), as previously described^42^. Apoptotic cells were stained with 10 μg/mL annexin V-fluorescein isothiocyanate (FITC) and 6 μg/mL PI and analysed via flow cytometry (FACSCalibur, Becton Dickinson, San Jose, CA).

### Cell transfection and shRNA-mediated gene silencing of p53

China Medical University committee has approved the experiments, including any relevant details. All experiments were performed in accordance with relevant guidelines and regulations. H1299 cells were transiently transfected with an empty vector (pcDNA3.1(-)), wild type (pcDNA3.1(-)-p53 WT), and mutant p53 (pCMV-Neo-Bam p53 R248W)^43^. Transfection agent with Polyjet^TM^ (SL100688; SignaGen Laboratories) was used per the manufacturer’s instructions. Expression levels were further confirmed with western blotting.

The specific lentiviral shRNA constructs targeted against TP53 were obtained from the National RNAi Core Facility (Institute of Molecular Biology, Genomic Research Center, Academia Sinica, Taiwan). The target sequences for TP53 were shTP53E (5′-CACCATCCACTACAACTACAT-3′). Lentivirus against TP53 was packaged in HEK293T cells and collected to infect Bm7 lung cancer cells as TP53 knockdown cells.

### Lipid raft detection

Cells were treated with phosphate-buffered saline (PBS) containing 0.53 mM ethylenediaminetetraacetic acid (EDTA), suspended in cold FACS buffer (0.1% foetal bovine serum in PBS), and then stained with either CTXB-FITC (12.5 μg/mL, SIGMA) for ganglioside M1 (GM1) or isotype immunoglobulin G (IgG)-FITC (Jackson) as a negative control for 30 minutes at 4 °C. Cells were washed with PBS and then examined via FACS analysis (FACSCalibur, BD). Untreated or simvastatin-treated cells were fixed with 4% paraformaldehyde, labelled with CTXB, and imaged with a Leica SP2 confocal microscope.

### Western blotting and co-immunoprecipitation

The following antibodies were used for western blotting: p53 (OP43; Merck Millipore), MDM2 (SMP14) (sc-965; Santa Cruz), AKT (2920; Cell Signaling Technology), phospho-AKT (Thr308) (9275; Cell Signaling Technology), phospho-AKT (Ser473) (9271; Cell Signaling Technology), PARP (9532; Cell Signaling Technology), caspase 3 (9661; Cell Signaling Technology), phospho-5′ AMP-activated protein kinase ([AMPK] bs-4002R; Bioss), AMPK (E-AB-30490; Elabscience Biotechnology Inc.), phospho-mammalian target of rapamycin ([mTOR] (E-AB-20929; Elabscience Biotechnology Inc.), mTOR (E-AB-32129; Elabscience Biotechnology Inc.), LC3A/B (4108; Cell Signaling Technology), α-tubulin (NB100-690; Novus Biologicals), GAPDH (10494-1-AP; Proteintech), HSP-40 (sc-59554; Santa Cruz Biotechnology), CHIP (sc-133066; Santa Cruz Biotechnology), and vinculin (GTX109749; GeneTex). For co-immunoprecipitation assay, cell lysates were incubated with p53 antibody (OP43, EMD Millipore, Billerica, MA) or HSP-40 antibody (sc-398766, Santa Cruz Biotechnology, Santa Cruz, CA) overnight at 4 °C. Then samples were precipitated with appropriate protein A/G beads with matched IgG as negative control. The precipitants were analysed in SDS-PAGE western blot.

### Time-lapse migration assay

A time-lapse migration assay was performed as previously described^44^. In brief, cells were cultured on dishes coated with collagen (10 μg/mL) and then treated with simvastatin. The accumulated distance was measured using the track point function of Image J software.

## Supplementary information

Supplementary Figure 1
