# Supplementary material for: Therapeutic effects of statins against lung adenocarcinoma via p53 mutant-mediated apoptosis
Source: Sci Rep. 2019 Dec 31;9:20403. doi: 10.1038/s41598-019-56532-6 (PMC6938497; doi:10.1038/s41598-019-56532-6)
Supplement: Supplementary file 1 — Supplementary Figure 1 [file 41598_2019_56532_MOESM1_ESM.pdf]

Therapeutic effects of statins against lung adenocarcinoma via p53 mutant-mediated apoptosis

Cheng-Wei Chou, Ching-Heng Lin, Tzu-Hung Hsiao, Chia-Chien Lo, Chih-Ying Hsieh, Cheng-Chung Huang, Yuh-Pyng Sher*

Supplementary information: Supplementary Figure 1.

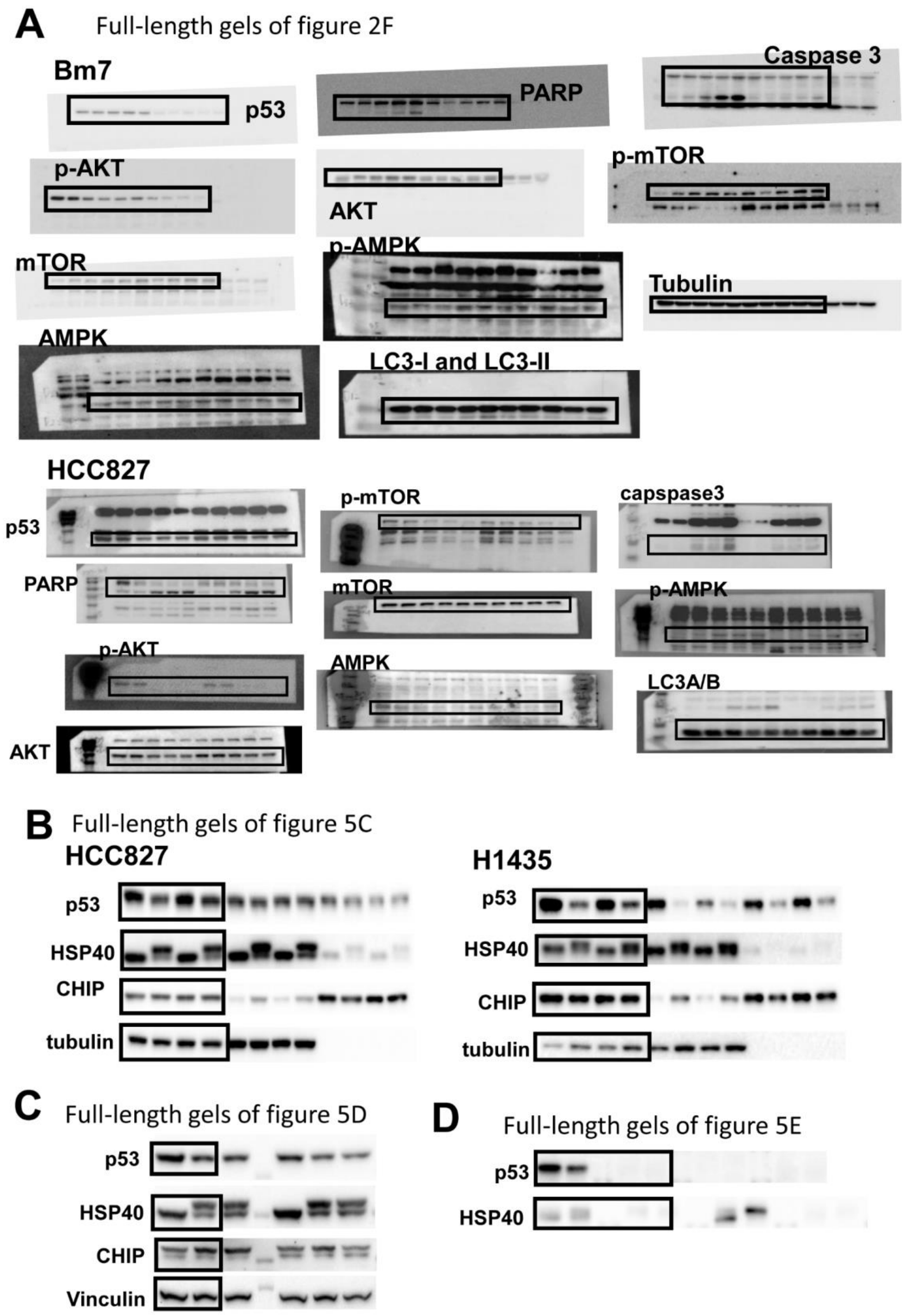

Supplementary Figure 1. (A) Full-length gels of figure 2F. (B) Full-length gels of figure 5C. (C) Full-length gels of figure 5D. (D) Full-length gels of figure 5E.
